# Associations between environmental perchlorate, nitrate, and thiocyanate exposure and severe headache or migraine: a cross-sectional, population-based analysis

**DOI:** 10.3389/fneur.2024.1431704

**Published:** 2024-10-24

**Authors:** Jiesheng Mao, Mi Zhou, Li Yanjun, Yunhan Zhao, Haoxiang Hu, Xiaokai Yang

**Affiliations:** Postgraduate Training Base Alliance of Wenzhou Medical University, Third Affiliated Hospital of Shanghai University, Wenzhou People’s Hospital, Wenzhou, China

**Keywords:** cross-sectional study, perchlorate, nitrate, thiocyanate, migraine, National Health and Nutrition Examination Survey

## Abstract

**Background:**

Environmental contaminants may play a significant role in the development of migraine. Perchlorate, nitrate, and thiocyanate were selected for this study due to their known impact on thyroid function, which is closely linked to neurological processes. Disruptions in thyroid function have been associated with various neurological disorders, including migraines. However, there is currently no evidence linking exposure to these specific chemicals to migraine. The study aims to evaluate the association between urinary concentrations of perchlorate, nitrate, and thiocyanate with the prevalence of severe headache or migraine in U.S. adults.

**Methods:**

A cross-sectional study was conducted using data from the National Health and Nutrition Examination Survey (NHANES) 2001–2004. Utilizing electrospray tandem mass spectrometry in conjunction with ion chromatography, urinary concentrations of perchlorate, nitrate, and thiocyanate urine were measured. Multiple logistic regression models were employed to evaluate the linear correlation between perchlorate, nitrate, and thiocyanate exposure and severe headache or migraine. The non-linear relationship is described analytically using a fitted smoothing curve and a two-piecewise regression model. Subgroup analyses were used to further clarify the stability of this relationship across different populations.

**Results:**

There were 1,446 participants in this population-based study, ranging in age from 20 to 85. After adjusting for potential confounding variables, the multiple logistic regression findings demonstrated that thiocyanate was significantly positively associated with the prevalence of migraine (odds ratio [OR] = 1.18; [1.06, 1.30]; *p* < 0.001). There was consistency in this connection across different subgroups (*p* for interaction >0.05). Furthermore, there was a non-linear correlation between urinary thiocyanate and migraine. Using a fitted smoothing curve and a two-piecewise regression model, it was found that the correlation between urinary thiocyanate and migraine was U-shaped (*p* for Log-likelihood ratio = 0.002). According to the findings of the multiple regression analysis, there was no significant correlation between urinary perchlorate and nitrate and migraine (both *p* > 0.05).

**Conclusion:**

We should limit our exposure to thiocyanate by keeping it within a reasonable range, as indicated by the U-shaped correlation between urinary thiocyanate and migraine.

## 1 Introduction

Severe headache or migraine are excruciating, disabling, and chronic neurovascular diseases. Patients with migraine usually present with moderately severe, unilateral throbbing headaches that can be exacerbated by exertion and are accompanied by nausea, vomiting, photophobia, and phonophobia (1). Around 1.04 billion people suffer from migraines globally. The prevalence in the US is around 18.2% for women and 6.5% for men, with a peak prevalence among adults between the ages of 25 and 55, placing a huge impact on personal mobility, healthcare systems, and society (2–4). Migraine patients often suffer from functional impairment caused by headache, such as limited activity, and normal inability to work, seriously affecting quality of life (5).

Although there are many medications that can be used to relieve migraine symptoms, there is currently no cure for migraine at its root. The factors linked to migraine in adults are not well elucidated, and a thorough investigation of migraine etiology is still lacking (6). The complex pathogenesis of migraine involves a combination of genetic predispositions and environmental triggers (7–10). Therefore, uncovering potential risk factors associated with migraine is essential for effective control and prevention strategies.

In recent years, growing concern has emerged regarding the potential impact of environmental pollutants on nervous system health, particularly in relation to migraines (11–13). Environmental toxins can exacerbate or trigger headache attacks through various mechanisms, such as inducing oxidative stress, disrupting nerve conduction, and initiating immune and inflammatory responses (14). The ongoing advancement of industrialization has led to the release of numerous chemical pollutants into the environment, among which perchlorate, nitrate, and thiocyanate are prevalent contaminants (15–18). These three chemicals can be detected in a variety of environmental media, such as wastewater and smoke, and have a wide range of health effects (19, 20). Urinary concentrations of these three toxicants are used as indicators of their exposure (21). Perchlorates, nitrates, and thiocyanates primarily act through a shared mechanism by inhibiting iodine uptake via sodium iodide symporters, which are responsible for transporting iodine to the vertebrate thyroid gland (22, 23). This inhibition can consequently lead to reduced thyroid hormone production. We speculate that these chemicals, which eventually lead to hypothyroidism after long-term suppression of iodine absorption, may have an impact on the nervous system, potentially increasing the risk of migraines (24). In previous studies, many scholars have explored the relationship between nitrate and migraine and further studied its mechanism, believing that nitrate is a common cause of migraine (25–27). A study from Malaysia found that communities with higher average levels of urinary thiocyanate had significantly higher rates of headache (28). However, there is still a lack of research on the correlation between perchlorate, nitrate, and thiocyanate exposure and migraines within the context of large population samples, leaving a significant gap in our understanding of their potential impact on migraine prevalence.

Through this cross-sectional study, we explored any potential association between urinary concentrations of perchlorate, nitrate, and thiocyanate and severe headache or migraine in the general adult population, aiming to provide new insights for migraine prevention.

## 2 Methods

### 2.1 Study population

**Figure 1:**
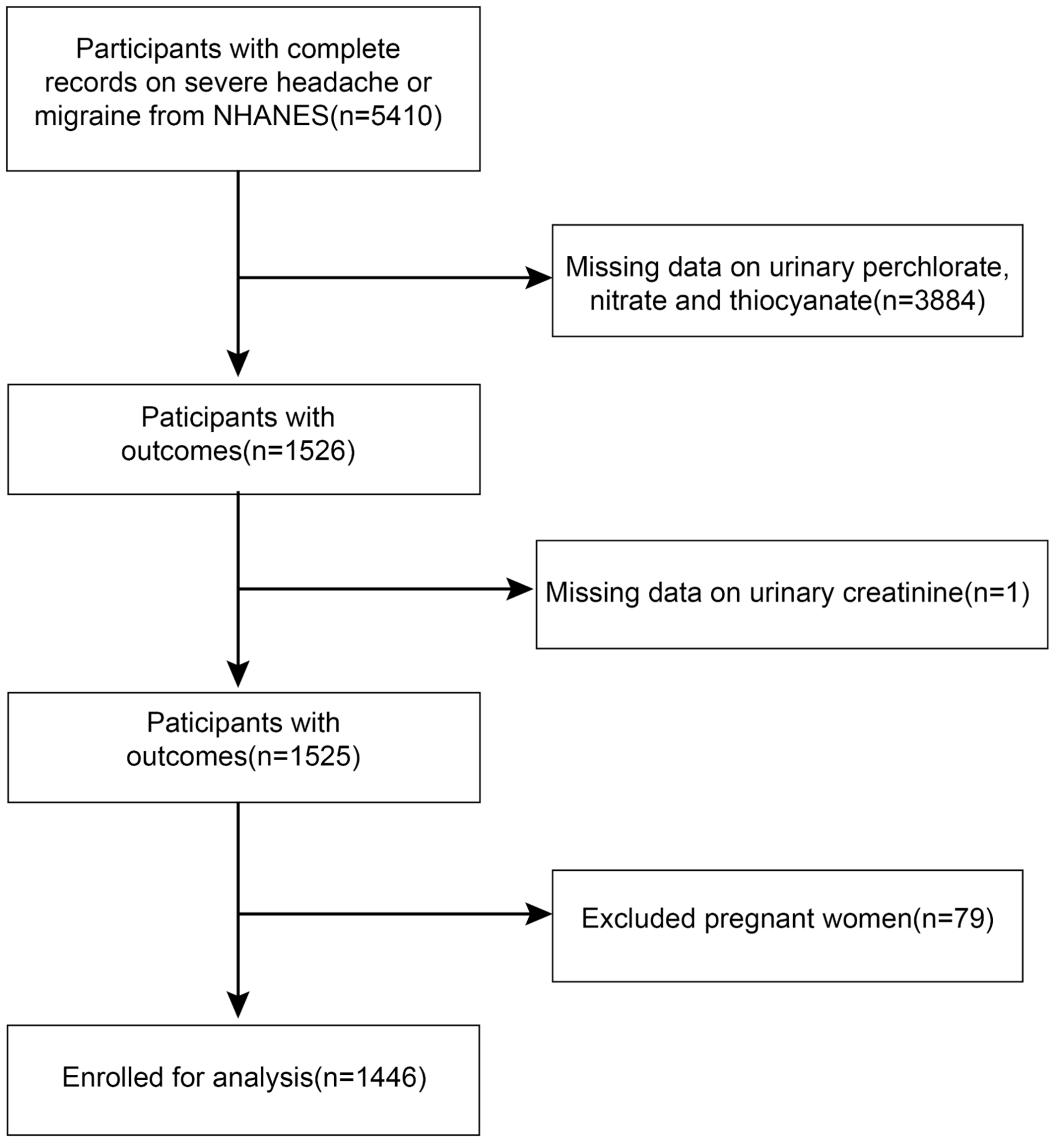
Flowchart of participants selection. NHANES, National Health and Nutrition Examination Survey.

This investigation utilized statistics from the National Health and Nutrition Examination Surveys (NHANES) database for the 2001–2004 cycle. The adults who took part in the survey were given a questionnaire task related to severe headaches or migraines, from which we obtained complete data on 5,410 participants. We ruled out pregnant women (*n* = 79) and individuals who lacked records for urinary perchlorate, nitrate, thiocyanate (*n* = 3,884), and urinary creatinine (*n* = 1). As a result, a total of 1,446 data sets were included in this study (Figure 1). Approval for the study was granted by the National Centre for Health Statistics Review Committee.

### 2.2 Exposure variable

Urine specimens were stored at −70°C to maintain the maximum purity of the samples. The specimens were extracted, and the ion content was measured by the National Center for Environmental Health. Using the sophisticated technique of ion chromatography-mass spectrometry, the concentrations of perchlorate, thiocyanate, and nitrate are analyzed in the sample (29). The relative response factors (ratio of natural analytes to stable isotope labeled internal standards) are compared to known standard concentrations to give the individual analyte concentrations. All assay results reported meet the specifications outlined in the Laboratory Sciences Department’s Quality Control and Quality Assurance Performance Standards for accuracy and precision.

### 2.3 Migraine assessment

Our methodology regarding migraine assessment is consistent with that used in previous studies (30–32). The NHANES questionnaire’s section on other types of pain included a self-report question about how severe a headache or migraine was. When asked if they had experienced a severe headache or migraine during the previous 3 months, those who responded “yes” were categorized as severe headache and migraine patients. We believe that the majority of individuals suffering from severe headaches had migraines, and the findings of the American Migraine Prevalence and Prevention (AMPP) supported our assumptions. According to the research, approximately 94.3% of participants complaining of severe headaches met the criteria of the International Classification of Headache Disoreder-2 (ICHDII-2) guideline for migraine or probable migraine (PM) (33). Therefore, we *characterized* participants as migraineurs regardless of whether they suffered from severe headaches or migraines. This diagnostic criterion is now widely used in current epidemiological studies (34, 35).

### 2.4 Covariates’ collection

Age, gender, race/ethnicity, educational attainment, and marital status were the demographic characteristics that we considered important. We included the participants’ energy, protein, and carbohydrate intakes separately in the Dietary Interview—Total Nutrient Intake list. We included body mass index (BMI) as an indicator to assess nutritional status, degree of fatness or leanness, or level of physical development. When BMI was used as a classification variable, less than or equal to 24 kg/m^2^ was defined as normal weight, greater than 24 kg/m^2^ and less than 28 kg/m^2^ was defined as overweight, and greater than or equal to 28 kg/m^2^ was defined as obese. We collected participants’ history of smoking and alcohol consumption as categorical variables. Regarding smoking history, participants who answered yes indicated that they had smoked at least 100 cigarettes throughout their lives, while those who answered no did not. Regarding drinking history, participants who answered yes indicated that they had had at least 12 bottles of any type of alcoholic beverage in a year, while those who answered no did not. In laboratory tests, we extracted data on serum cholesterol concentration and inflammatory marker C-reactive protein. Data on the patient’s significant disease history were collected by answering the questionnaire “Have you ever been informed by a physician or other healthcare provider that you have diabetes, high blood pressure, or a stroke?” Considering that perchlorates, nitrates and thiocyanates can affect thyroid function, we adjust thyroxine. For the measurement of urinary biomarkers, urinary creatinine was taken into account in order to control for measurement error bias due to urinary dilution. These are all the covariates that we included in our consideration (36).

### 2.5 Statistical analysis

Our statistics present continuous variables as mean (standard deviation) or median [lower quartile, upper quartile], while categorical variables are presented as numbers (percentage). Chi-square tests, Mann–Whitney U-tests, and two-sample t-tests were used to examine group differences. Because the statistics were non-normally distributed, a log_2_-transformation scheme was employed for the statistics for perchlorate, nitrate, and thiocyanate in urine in order to make the data more applicable to the scenarios in which our statistical methods were used (37). The statistical analysis was performed with the help of R-project (version 4.2) and EmpowerStats (version 4.1), both of which are computer programs that specialize in statistical computing and visuals. In order to provide data that is representative of the U.S. population, all estimates are therefore calculated using sample weights in accordance with NCHS’s analytical guidelines. A weighted multiple logistic regression model was utilized for the purpose of investigating the connection that exists between urinary concentrations of thiocyanate, nitrate, and perchlorate with the prevalence of migraine. To explore the relationship’s non-linearity, generalized additive models and smoothed curve fits were utilized. We employed a two-piece regression model in order to further investigate the non-linear correlations and determine the inflection points. The log-likelihood ratio test was utilized to figure out whether there was a threshold effect of exposure factors on the outcome variable. Subgroup analysis and interaction tests were conducted to explore whether the relationship was modified by sex, age, race, BMI, and disease history of hypertension or diabetes. Statistical significance was determined by *p* < 0.05.

## 3 Results

### 3.1 Baseline characteristics

Based on the inclusion and exclusion criteria, 1,446 adults were included in the study, and their average age was 50.02 ± 18.71 years. Men made up 50.07% of these participants, while women made up 49.93%. Median values of perchlorate, nitrate, and thiocyanate levels in urine were 3.80 (2.10 [first quartile], 6.30 [third quartile]) μg/L, 48.00 (28.0–78.00) mg/L, and 1,400 (620–3,000) μg/L, accordingly. Among all the respondents, there were 308 headache cases. Between the headache group and the non-headache group, differences in age, gender, ethnicity, and urinary thiocyanate levels were statistically significant (all *p* < 0.05). In contrast to the non-headache group, respondents from the headache group were younger, had a greater percentage of females, a greater percentage of Mexican Americans and non-Hispanic black persons, and were more inclined to have elevated urinary thiocyanate levels. The difference between the two groups in urinary perchlorate and nitrate failed to reach statistical significance (both *p* > 0.05) (Table 1).

**Table 1:** Characteristics of 1,446 participant aged 20–85 years from 2001 to 2004 National Health and Nutrition Examination Survey (NHANES).

Characteristic	Non-headache (*n* = 1,138)	Headache (*n* = 308)	*p* value
Age, years	51.72 (19.2)	43.70 (15.1)	<0.001
Sex (%)			<0.001
Male	602 (52.9)	122 (39.6)	
Female	536 (47.1)	186 (60.4)	
Race (%)			0.010
Non-Hispanic white person	636 (55.9)	149 (48.4)	
Non-Hispanic black person	205 (18.0)	62 (20.1)	
Mexican American	225 (19.8)	62 (20.1)	
Others	272 (6.3)	35 (11.4)	
Marital status (%)			0.133
Married or living with a partner	722 (63.4)	181 (58.8)	
Living alone	416 (36.6)	127 (41.2)	
Education level (%)			0.089
< High school	339 (29.8)	110 (35.7)	
High school or GED	262 (23.0)	72 (23.4)	
>High school	537 (47.2)	126 (40.9)	
Smoked at least 100 cigarettes in life	572 (50.3)	167 (54.2)	0.408
Had at least 12 alcoholic drinks/year	755 (66.3)	191 (62.0)	0.296
Hypertension, %	366 (32.2)	91 (29.6)	0.648
Diabetes, %	109 (9.6)	24 (7.8)	0.169
Stroke, %	42 (3.7)	12 (3.9)	0.866
BMI, kg/m^2^	28.0 (5.9)	28.1 (6.2)	0.828
C reactive protein, mg/dL	0.4 (0.8)	0.3 (0.4)	0.590
Total cholesterol, mg/dL	204.3 (43.4)	199.9 (40.0)	0.058
Energy, kcal/day	2102.2 (932.1)	2155.7 (1026.0)	0.643
Protein intake, g/day	78.91 (38.5)	80.0 (43.84)	0.837
Carbohydrate intake, g/day	263.0 (123.4)	273.6 (138.5)	0.494
Creatinine, mg/dL	125.1 (84.0)	132.0 (84.9)	0.160
Thyroxine, nmol/L	105.4 (24.9)	105.5 (23.3)	0.749
Perchlorate, μg/L	3.9 [2.1, 6.3]	3.5 [2.1, 6.1]	0.332
Nitrate, mg/L	48.0 [27.0, 77.0]	50.5 [33.0, 78.0]	0.144
Thiocyanate, μg/L	1,300 [590, 2,600]	1,900 [740, 5,000]	<0.001

Data are presented as *n* (%) and mean (SD) or median (lower quartile, upper quartile). BMI, body mass index.

### 3.2 Association between urinary thiocyanate and severe headache or migraine

For the purpose of examining the linearity or association between exposure and migraine, we computed the OR for every quartile of exposure (Table 2). Urinary concentrations of perchlorate and nitrate did not meet significant relevance to migraine, as demonstrated in Table 2. However, the present results showed a link between a higher concentration of urinary thiocyanate with an increased prevalence of migraine. The urinary thiocyanate was positively correlated with the prevalence of migraine throughout the three models. (Model 1: OR = 1.19, 95% CI: 1.09–1.30, *p* < 0.001; Model 2: OR = 1.17, 95% CI: 1.07–1.29, *p* < 0.001; and Model 3: OR = 1.18, 95% CI: 1.06–1.30, *p* < 0.001). Once the potential confounders have been taken into account within the multiple logistic regression, the highest quartile (Q4) remained significantly associated with migraine in comparison to the lowest quartile (Q1) of urinary thiocyanate (*p* < 0.001 for Model 1 and Model 2, *p* < 0.05 for Model 3).

**Table 2:** Association of urinary perchlorate, nitrate, and thiocyanate with headache.

Subgroup	Cases	*N*	Model 1		Model 2		Model 3	
			OR [95%Cl]	*p*	OR [95%Cl]	*p*	OR [95%Cl]	*p*
Perchlorate								
Q1 (≤1.58)	74	357	Ref		Ref		Ref	
Q2 (1.63–2.23)	93	358	1.41 [0.98, 2.05]	0.068	1.53 [1.05, 2.25]	0.028	1.52 [1.02, 2.06]	0.038
Q3 (2.26–2.85)	67	366	0.90 [0.60, 1.34]	0.594	0.94 [0.62, 1.43]	0.785	0.91 [0.59, 1.40]	0.667
Q4 ≥ 2.87	74	365	1.01 [0.67, 1.53]	0.967	1.04 [0.68, 1.59]	0.871	1.03 [0.65, 1.63]	0.909
Log2	308	1, 446	0.94 [0.81, 1.09]	0.426	0.95 [0.81, 1.11]	0.497	0.92 [0.78, 1.10]	0.359
Nitrate								
Q1 (≤4.81)	63	351	Ref		Ref		Ref	
Q2 (4.86–5.58)	83	358	1.32 [0.89, 1.96]	0.161	1.36 [0.91, 2.03]	0.134	1.27 [0.84, 1.92]	0.265
Q3 (5.61–6.29)	83	374	1.21 [0.79, 1.85]	0.378	1.23 [0.80, 1.90]	0.354	1.04 [0.65, 1.66]	0.874
Q4 ≥ 6.30	79	363	1.11 [0.69, 1.80]	0.669	1.06 [0.64, 1.74]	0.829	0.83 [0.47, 1.45]	0.517
Log2	308	1, 446	1.08 [0.93, 1.26]	0.325	1.07 [0.91, 1.25]	0.419	1.00 [0.83, 1.19]	0.967
Thiocyanate								
Q1 (≤9.25)	62	357	Ref		Ref		Ref	
Q2 (9.28–10.34)	57	363	0.87 [0.58, 1.31]	0.496	0.91 [0.60, 1.38]	0.662	0.91 [0.60, 1.39]	0.665
Q3 (10.45–11.50)	76	362	1.33 [0.89, 1.99]	0.162	1.31 [0.91, 2.08]	0.131	1.38 [0.91, 2.11]	0.132
Q4 ≥ 11.55	113	364	2.09 [1.40, 3.11]	< 0.001	2.05 [1.32, 3.18]	0.001	2.04 [1.29, 3.24]	0.002
Log2	308	1, 446	1.19 [1.09, 1.30]	<0.001	1.17 [1.07, 1.29]	<0.001	1.18 [1.06, 1.30]	<0.001

CI, confidence interval; OR, odds ratio. Q1–Q4, Quartiles based on urinary perchlorate, nitrate, and thiocyanate concentrations.

Model 1: adjusted for creatinine, age, gender, and race.

Model 2: Model 1+ marital status, education level, BMI, drinking, smoking, stroke, hypertension, diabetes, C reactive protein, thyroxine, total cholesterol, energy, protein intake, carbohydrate intake.

Model 3: Model 2+ mutual adjustment of log2-transformed urinary perchlorate, nitrate, and thiocyanate.

**Figure 2:**
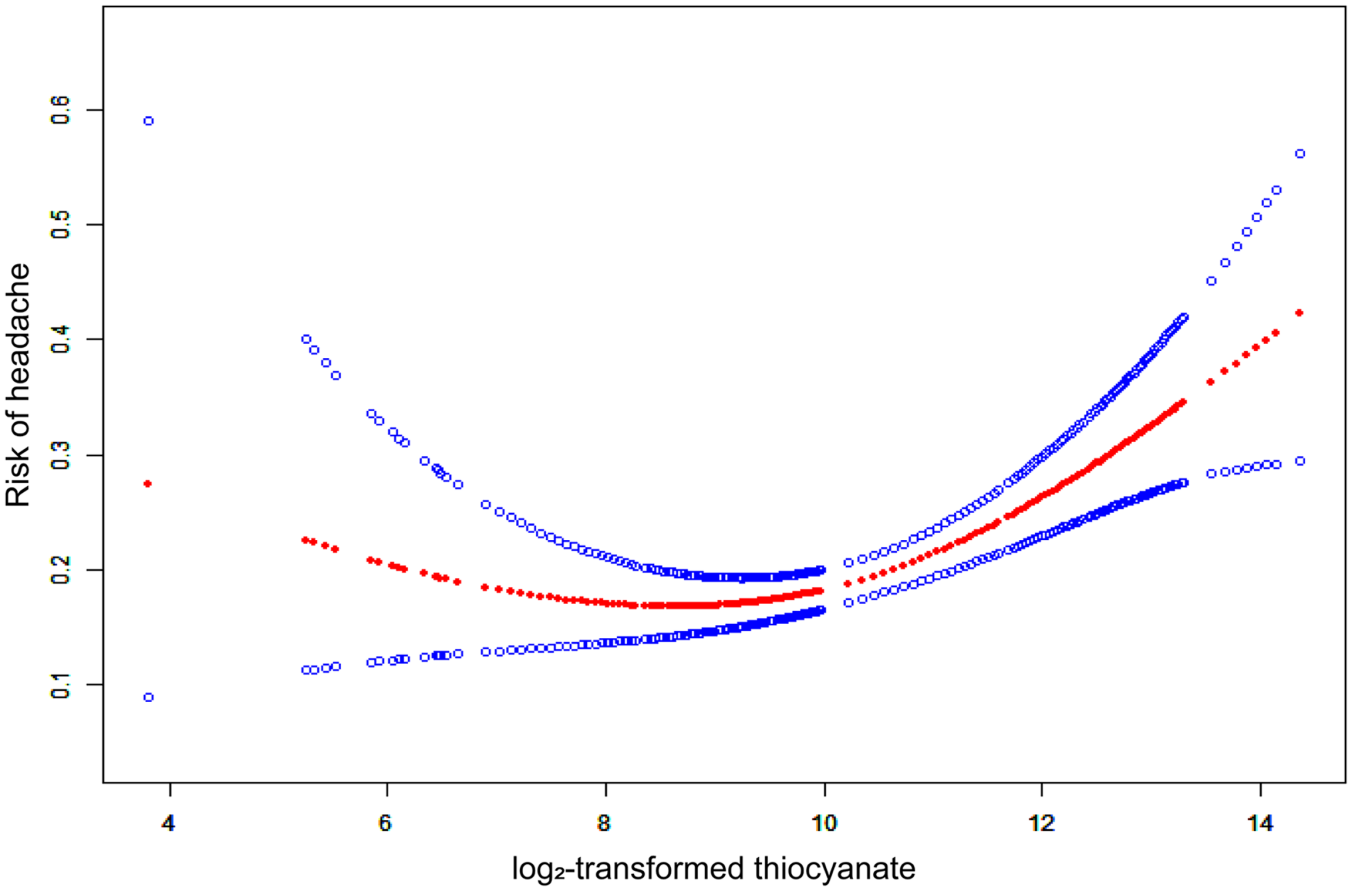
The non-linear associations between thiocyanate and migraine. The solid red line represents the smooth curve fit between variables. Blue bands represent the 95% confidence interval from the fit.

**Figure 3:**
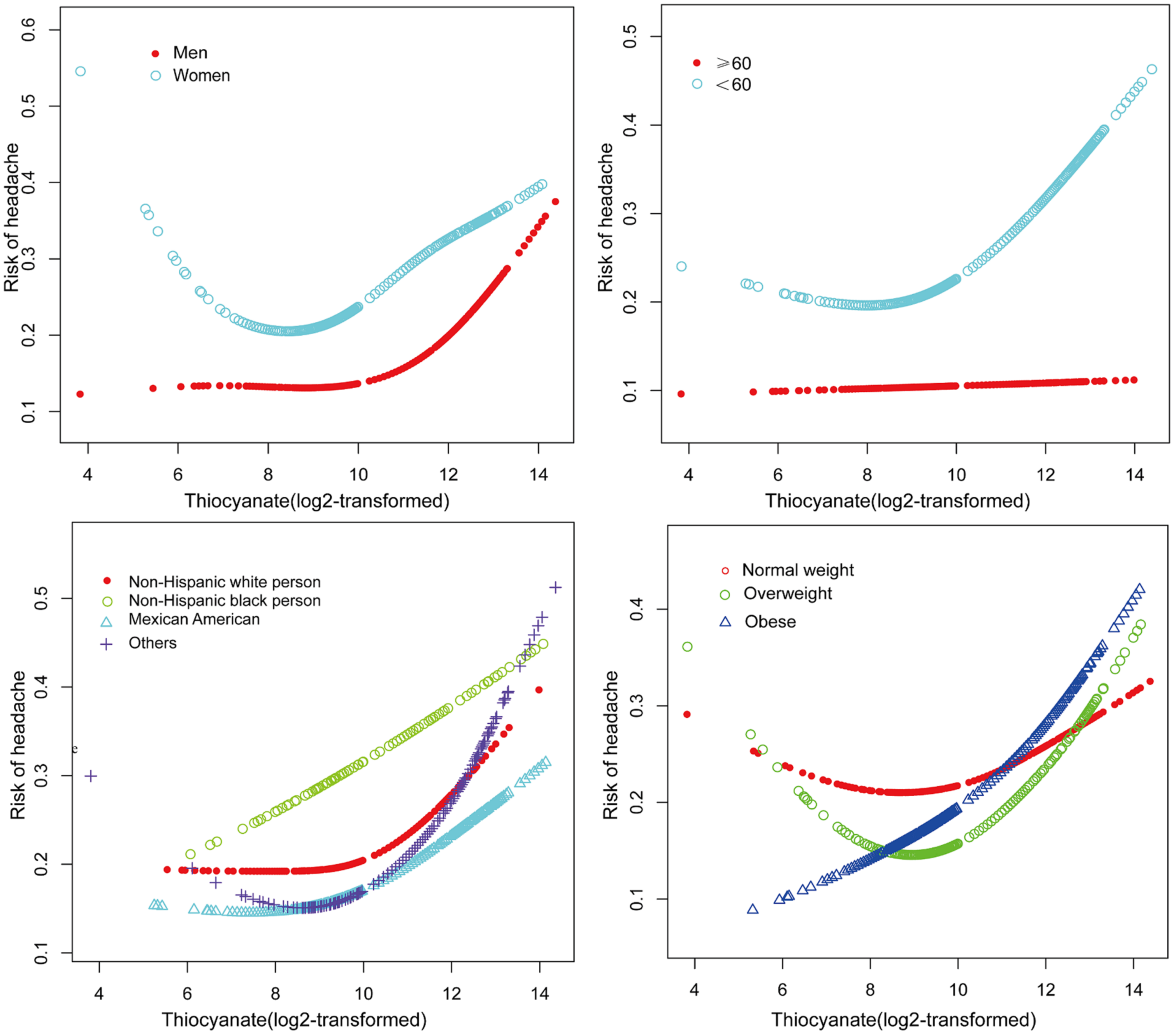
The non-linear associations between thiocyanate and migraine Stratified by gender, age, race, and BMI.

We fitted a smooth curve to depict the non-linear correlation between thiocyanate and migraine (Figure 2). A U-shaped relationship between urinary thiocyanate (log_2_-transformed) and the prevalence of migraine was discovered employing a two-piecewise regression model, with an inflection point of 9.23 μg/L (Table 3). After we stratified our analyses by age, sex, race, and BMI, we found that this u-shaped association was also present in women, younger than 60 years, non-Hispanic white persons and other races, and overweight people (Figure 3). In threshold effects analyses, we further found their inflection points to be 7.49, 8.64, 10.45, 9.37, and 8.23 (Table 3).

**Table 3:** Threshold effect analysis of thiocyanate on headache.

Headache	OR [95%Cl] *p*
Thiocyanate (log_2_-transformed)	
Total	
Inflection point	9.23
Thiocyanate (log_2_-transformed) <9.23	0.82 [0.65, 1.03] 0.083
Thiocyanate (log_2_-transformed) >9.23 μg/L	1.33 [1.17, 1.50] < 0.001
P for Log-likelihood ratio	0.002
Women	
Inflection point	7.49
Thiocyanate (log_2_-transformed) < 7.49	0.52 [0.28, 1.00] 0.048
Thiocyanate (log_2_-transformed) > 7.49	1.23 [1.06, 1.42] 0.007
P for Log-likelihood ratio	0.019
Below 60 years	
Inflection point	8.64
Thiocyanate (log_2_-transformed) < 8.64	0.68 [0.46, 1.00] 0.049
Thiocyanate (log_2_-transformed) > 8.64	1.35 [1.18, 1.55] < 0.001
P for Log-likelihood ratio	0.003
Non-Hispanic White person	
Inflection point	10.45
Thiocyanate (log_2_-transformed) < 10.45	0.88 [0.60, 1.29] 0.510
Thiocyanate (log_2_-transformed) > 10.45	2.00 [1.14, 3.50] 0.015
P for Log-likelihood ratio	0.040
Others	
Inflection point	9.37
Thiocyanate (log_2_-transformed) < 9.37	0.67 [0.42, 1.04] 0.077
Thiocyanate (log_2_-transformed) > 9.37	1.75 [1.25, 2.45] < 0.001
P for Log-likelihood ratio	0.007
Overweight	
Inflection point	8.23
Thiocyanate (log_2_-transformed) < 8.23	0.40 [0.21, 0.77] 0.006
Thiocyanate (log_2_-transformed) > 8.23	1.50 [1.19, 1.89] < 0.001
*P* for Log-likelihood ratio	0.001

Age, gender, race, marital status, education level, BMI, drinking, smoking, stroke, hypertension, diabetes, C reactive protein, thyroxine, total cholesterol, energy, protein intake, carbohydrate intake, creatinine, thyroxine, log_2_-transformed urinary perchlorate, and log_2_-transformed nitrate were adjusted. CI, confidence interval; OR, odds ratio.

### 3.3 Subgroup analysis

**Figure 4:**
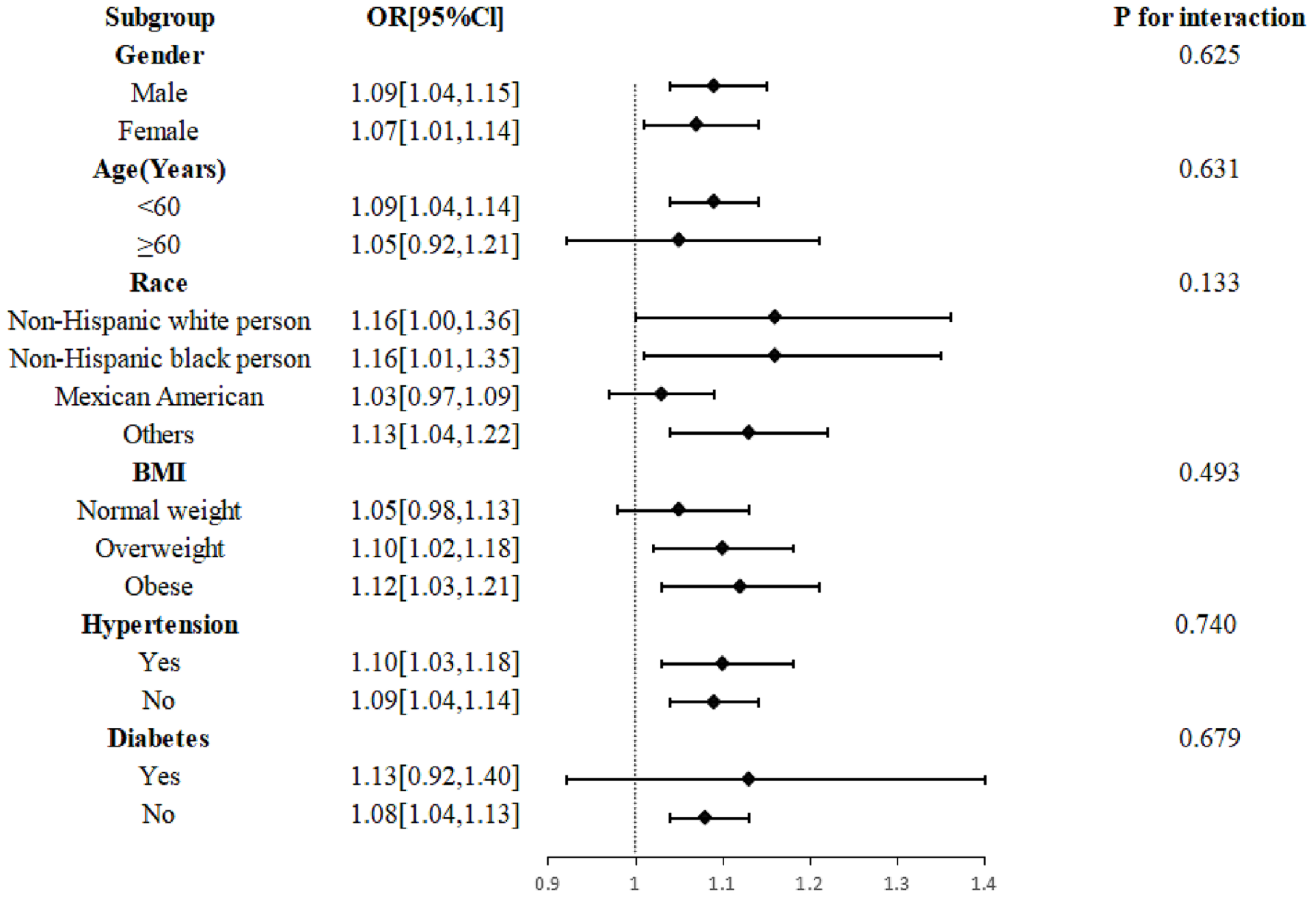
Subgroup analysis of the effect of thiocyanate on migraine. Age, gender, race, marital status, education level, BMI, drinking, smoking, stroke, hypertension, diabetes, C reactive protein, total cholesterol, energy, protein intake, carbohydrate intake, creatinine, thyroxine, log2-transformed urinary perchlorate, and log2-transformed nitrate were adjusted. BMI, body mass index; CI, confidence interval; OR, odds ratio.

The outcomes of the study suggested that the association between urinary thiocyanate with headache was consistent. There was no significant interaction even though this association was stronger in participants under 60 years old, non-Hispanic Black persons and other races, overweight and obese patients, and those without diabetes (Figure 4).

## 4 Discussion

In our research groups, which are nationally representative of adults from the US, urinary thiocyanate was positively related to migraine, and there were no significant associations between urinary perchlorate and nitrate and migraine. Notably, we observed a U-shaped correlation between urinary thiocyanate concentrations and migraine, with a statistically significant inflection point of 9.23 μg/L. As far as we are aware, our study suggests for the first time that urinary thiocyanate is a risk factor for migraine. Our research puts forward an indicative target and rationale for urinary thiocyanate regulation in migraineurs.

Severe headache has been reported as a common adverse effect of nitrate drugs, and nitrates may play a very important role in this (38, 39). Antonio et al. conducted a cohort study and observed that nitrate levels were significantly higher in samples of migraineurs than in non-migraineurs (27). However, our study showed that although nitrate levels were higher in the headache group than in the non-headache group, the data were not statistically significantly different, and further sample expansion is still needed to verify the relationship in subsequent studies. Also, our study yielded no statistical association between perchlorate concentrations and migraine. Early investigations have discovered that factors associated with migraine include dietary factors such as calcium, magnesium, iron, and zinc (30, 31, 34), emotional factors related to optimism or pessimism (35), sex hormone levels (40), and air pollution exposure (41). Our investigation adds a novel contribution that thiocyanate exposure is associated with migraine. Noor et al. conducted a cross-sectional study of population cyanide exposure in communities near gold mines and showed that mean urinary thiocyanate levels were significantly higher in exposed communities than in control communities, along with a significantly higher prevalence of headaches in exposed populations (28). This is consistent with the findings of our study. In contrast, our investigation enrolled a relatively large population sample and performed smooth curve fitting to further characterize the non-linear relationship between thiocyanate and migraine. What’s more interesting, we discovered a U-shaped correlation between urinary thiocyanate (log_2_-transformed) and migraine prevalence. This means that if thiocyanate exposure is controlled at a reasonable level, the prevalence of migraines can be reduced.

The mechanism of action of toxins in headache attacks may be related to their multifaceted effects on the nervous system. First, many environmental toxins, such as heavy metals, pesticides, and air pollutants, have been shown to increase oxidative stress in the body, a state of stress that may exceed the capacity of antioxidant defense mechanisms, leading to neuronal damage and inflammatory responses (42). Oxidative stress is not only associated with the onset of migraine, but also may aggravate headache by affecting the trigeminal vascular system. Another possible mechanism involves the toxin-triggered immune response and inflammatory cascade. Airborne pollutants, such as fine particulate matter (PM2.5) and ozone, have been shown to activate immune cells and release pro-inflammatory cytokines such as tumor necrosis factor-*α* (TNF-α) and interleukin-6 (IL-6), which in the central nervous system can cause sensitization of neurons and enhancement of pain (43).

Despite the fact that the mechanisms underlying thiocyanate’s effects on migraine disease are not fully understood, our findings are in agreement with the available data. It is of great significance to further explore and study the mechanisms involved. Superoxide dismutase, a key antioxidant enzyme, is the only element identified to be consistently decreased in migraineurs (44). It has been shown that thiocyanate and other anions bind to copper-containing superoxide dismutase (SOD) at the copper and zinc sites of the natural enzyme (Cu_2_Cu_2_SOD). Electron spin resonance spectroscopy indicates that the binding of SCN^−^ to Cu_2_Cu_2_SOD disrupts the imidazole ester bridge between the two copper centers. The thiocyanate replaces the bridging histidine of the Cu^2+^ ion in the copper site and also replaces the aspartate ligand of the Cu^2+^ ion in the zinc site. When SCN binds to Cu^2+^, the imidazole acid bridge between the two copper ions is broken. This has an effect on superoxide dismutase action and may contribute to oxidative stress (45). Secondly, myeloperoxidase (MPO) catalyzed thiocyanate oxidation produces cyanate of endogenous origin (46). According to recent research, cyanate may attack vascular tissue by producing an excessive amount of reactive oxygen species (ROS) (47). Cyanate can induce oxidative stress injury by inhibiting the Nrf2/HO-1 pathway (47). Migraine occurs when the human body system is unable to effectively balance the effects of reactive oxygen molecules. Furthermore, thiocyanate exposure usually induces a high inflammatory response (48, 49). A cohort study showed that thiocyanate exposure induces allergic inflammation, which is associated with allergy-related symptoms (48). Thiocyanates may exacerbate inflammatory responses in autoimmune and gastrointestinal diseases (49, 50). Additionally, thiocyanates can affect thyroid function by inhibiting iodine absorption over time, which in turn can impact the nervous system, brain energy metabolism, and neurotransmitter regulation due to the crucial role of thyroid hormones in these processes (23, 24). Changes in thyroid function may lead to increased sensitivity in the nervous system, which in turn increases susceptibility to migraines. Therefore, its effect on migraine may be related to the role of various cytokines in the inflammatory process or the combination of other diseases. However, further investigation in prospective longitudinal studies is needed to examine the connection between thiocyanate exposure and migraine.

It is worth mentioning that the main adverse effects of sodium nitroprusside, a commonly used drug in the clinical treatment of hypertensive emergencies and acute left heart failure, are hypotension and headache (51). Song et al. described it as a headache trigger (52). Enoch et al. did an in-depth study on the mechanism of action (53). Since the metabolite of sodium nitroprusside is cyanide and the final metabolite is thiocyanate, in view of our study, whether its toxic effects in migraine can be associated with thiocyanate accumulation deserves further study and investigation, which has important clinical implications.

This study has several strengths. Firstly, this study utilized NHANES data, which are population-based sampling data gathered nationwide using a predetermined procedure. Since the appropriate NHANES sampling weights were taken into account in all analyses, the study samples were more representative. Moreover, the exploration of non-linear relationships and multiple adjustments, as well as subgroup analysis, improved the reliability of the results. However, the restrictions of this research should not be neglected. Due to the nature of the cross-sectional study, the authors were unable to obtain a clear causal relationship. Secondly, a single experimental data of thiocyanate may not accurately reflect long-term exposure. Although the records are founded on a nationally representative dataset, the data used by the authors were collected from 2001 to 2004, almost 20 years ago. The authors attempted to analyze this association using more recent data, but data on questionnaire collection for headache as well as data on urinary thiocyanates, were only available concurrently in the NHANES 2001–2004 cycle. Considering that exposure levels of environmental pollutants may change over time, as well as potential changes in lifestyle and socioeconomic factors, the long-term environmental exposure effects of these chemicals will need to be analyzed in the future with data from different time spans. Finally, the results of this study, severe headache or migraine, were based on self-report and may be subject to recall bias or subjectivity. We omitted more detailed information about the frequency and severity of headaches. This may lead to biased results, fail to identify specific risk factors for different headache subtypes, and limit multilevel or more complex statistical analyses (e.g., dose–response relationship analyses). In future studies, we suggest using more refined analyses to explore the relationship between exposure levels and headache frequency and associated disability severity to reveal the underlying mechanisms more comprehensively.

## 5 Conclusion

The present study established a U-shaped correlation between thiocyanate exposure and migraine in a US adult population. These results suggest the importance of rational management and regulation of chemicals in the environment for the prevention of migraine in adults. Further studies are needed to focus on practical strategies for monitoring exposure levels and potential mechanisms for the association between exposure and migraine.

## Data Availability

The datasets presented in this study can be found in online repositories. The names of the repository/repositories and accession number(s) can be found in the article/supplementary material.
